# From eye to arrow: Attention capture by direct gaze requires more than just the eyes

**DOI:** 10.3758/s13414-021-02382-2

**Published:** 2021-11-02

**Authors:** Christina Breil, Lynn Huestegge, Anne Böckler

**Affiliations:** 1 Julius-Maximilians-University of Würzburg, Würzburg, Germany; 2 Department of Psychology III, University of Würzburg, Röntgenring 11, 97070 Würzburg, Germany; 3 Leibniz University Hannover, Hannover, Germany; 4 Max-Planck-Institute for Human Cognitive and Brain Sciences, Leipzig, Germany

**Keywords:** Social cognition, Attention capture, Direct gaze, Social cues, Face perception, Social interaction

## Abstract

**Abstract:**

Human attention is strongly attracted by direct gaze and sudden onset motion. The sudden direct-gaze effect refers to the processing advantage for targets appearing on peripheral faces that suddenly establish eye contact. Here, we investigate the necessity of social information for attention capture by (sudden onset) ostensive cues. Six experiments involving 204 participants applied (1) naturalistic faces, (2) arrows, (3) schematic eyes, (4) naturalistic eyes, or schematic facial configurations (5) without or (6) with head turn to an attention-capture paradigm. Trials started with two stimuli oriented towards the observer and two stimuli pointing into the periphery. Simultaneous to target presentation, one direct stimulus changed to averted and one averted stimulus changed to direct, yielding a 2 × 2 factorial design with direction and motion cues being absent or present. We replicated the (sudden) direct-gaze effect for photographic faces, but found no corresponding effects in Experiments 2–6. Hence, a holistic and socially meaningful facial context seems vital for attention capture by direct gaze.

**Statement of significance:**

The present study highlights the significance of context information for social attention. Our findings demonstrate that the direct-gaze effect, that is, the prioritization of direct gaze over averted gaze, critically relies on the presentation of a meaningful holistic and naturalistic facial context. This pattern of results is evidence in favor of early effects of surrounding social information on attention capture by direct gaze.

## Introduction

Faces are special to us. In our everyday lives, we encounter a vast amount of information that is relevant for our well-being, yet we exhibit a striking susceptibility for facial configurations from early on (Goren et al., 1975). One of the first and most frequently fixated regions within the human face are the eyes (Arizpe et al., 2017). Eye gaze conveys essential information about attentional, intentional, and emotional states and is indispensable for social communication (Schilbach, 2015; Tomasello & Carpenter, 2007). Accordingly, we are specialized in detecting the direction of another’s attention, and human eyes with their white sclera seem particularly effective in conveying this information (Emery, 2000). Neurons in the superior temporal sulcus of monkeys and humans specifically respond to the direction of the eyes (Perret et al., 1985), and several facial features further emphasize the salience of this region (Emery, 2000).

A well-known effect in the social attention literature is “gaze following,” the finding that we rapidly shift attention according to others’ gaze direction, resulting in a processing advantage for this location (Driver et al., 1999; Friesen & Kingstone, 1998; Frischen et al., 2007). In addition, humans are extraordinarily sensitive to direct gaze. Researchers have proposed that direct gaze immediately activates sub-cortical structures and facilitates subsequent cognitive and perceptual processing (Senju & Johnson, 2009). Even though, under some circumstances such as very brief or masked presentations, a processing advantage for averted gaze has also been reported (e.g. Riechelmann et al., 2020), direct gaze seems to be preferred over averted gaze from childhood on (Farroni et al., 2004) and also constitutes a magnet for human attention in adulthood (Mojzisch et al., 2006; Palanica & Itier, 2012). We detect a specific face among other faces faster when it directly looks at us, which has been labeled as the “stare-in-the-crowd-effect” (Doi et al., 2009; Palanica & Itier, 2011; Von Grünau & Anston, 1995). Moreover, discriminating direct from averted gaze is still accurate when a second task is performed concurrently, whereas discriminating averted left from averted right gaze suffers from dual-task demands (Yokoyama et al., 2014).

Attentional capture by direct gaze is particularly pronounced when eye contact co-occurs with sudden-onset motion of the face, two cues that seem to influence information processing additively and in parallel (Böckler et al., 2014). In this task, participants identified targets that were presented on the forehead of one of four face images in a 2 × 2 within-subjects design, with gaze direction (direct or averted) and apparent face motion (static or sudden) as within-subject factors. With this initial combination of gaze and motion cues, Böckler et al. (2014) found that targets are classified faster when they were presented on faces that suddenly established eye contact (sudden direct-gaze effect).

Until now, the sudden direct-gaze effect has been investigated exclusively with images of real faces. In contrast to gaze following research, where effects of a variety of social and non-social ostensive stimuli (such as arrows) have been systematically addressed and demonstrated (Friesen & Kingstone, 1998; Frischen et al., 2007; Hietanen & Yrttimaa, 2005; Ristic et al., 2002; Tipples, 2005), the role of particular stimulus features on attention capture by direct gaze remains unknown. Generalizing findings from one paradigm to the other is, however, precarious due to fundamental differences between them: While cues are centrally presented and specifically attended to in gaze cueing, stimuli appear in the periphery and serve as distractors in our task. At the current state of research, one cannot estimate the extent to which the observed effect relies on direct *gaze* at all as compared to the mere feeling of “being addressed.” Here, we present an experimental series that is specifically designed to close this gap. We probe whether and to what degree the sudden direct-gaze effect relies on naturalistic and holistic social information. Specifically, we ran the attention capture paradigm by Böckler et al. (2014) with six different sets of stimuli: photographs of real faces (photographic and holistic face information) similar to the original study, arrows (no social but directional information), isolated schematic eyes (absence of both photographic and holistic face information), photographs of isolated eyes (photographic; no holistic face information), and schematic faces (holistic; no photographic face information). Following the notion that congruence of head and eye orientation shapes the detection of gaze direction (Conty et al., 2006), two versions of schematic faces were employed: One with frontal head view in all experimental conditions (*no head turn*; Experiment 5) and one switching between frontal and deviated head view between conditions, hence creating the impression of a head-turn movement similar to the one in Experiment 1 (Experiment 6). A feature that is common to all experiments (probably except for arrows) is the ostensive signal of being addressed: the stimulus is either targeted towards the observer or into the periphery. This setup hence manipulated the degree to which holistic and photographic social information was provided and allowed targeting the boundary conditions that enable attention capture by direct gaze. Arrow stimuli were implemented in order to directly compare social with directional information.

We hypothesized that the gaze effect would decrease together with the level of holistic and naturalistic social information. Specifically, we expected the strongest gaze effect with photographs of real faces (Experiment 1) and attenuated or absent gaze/direction effects for arrows (Experiment 2), isolated eyes (Experiments 3 and 4), and schematic faces (Experiments 5 and 6).

## Materials and methods

### Experimental setup and procedure

**Fig. 1:**
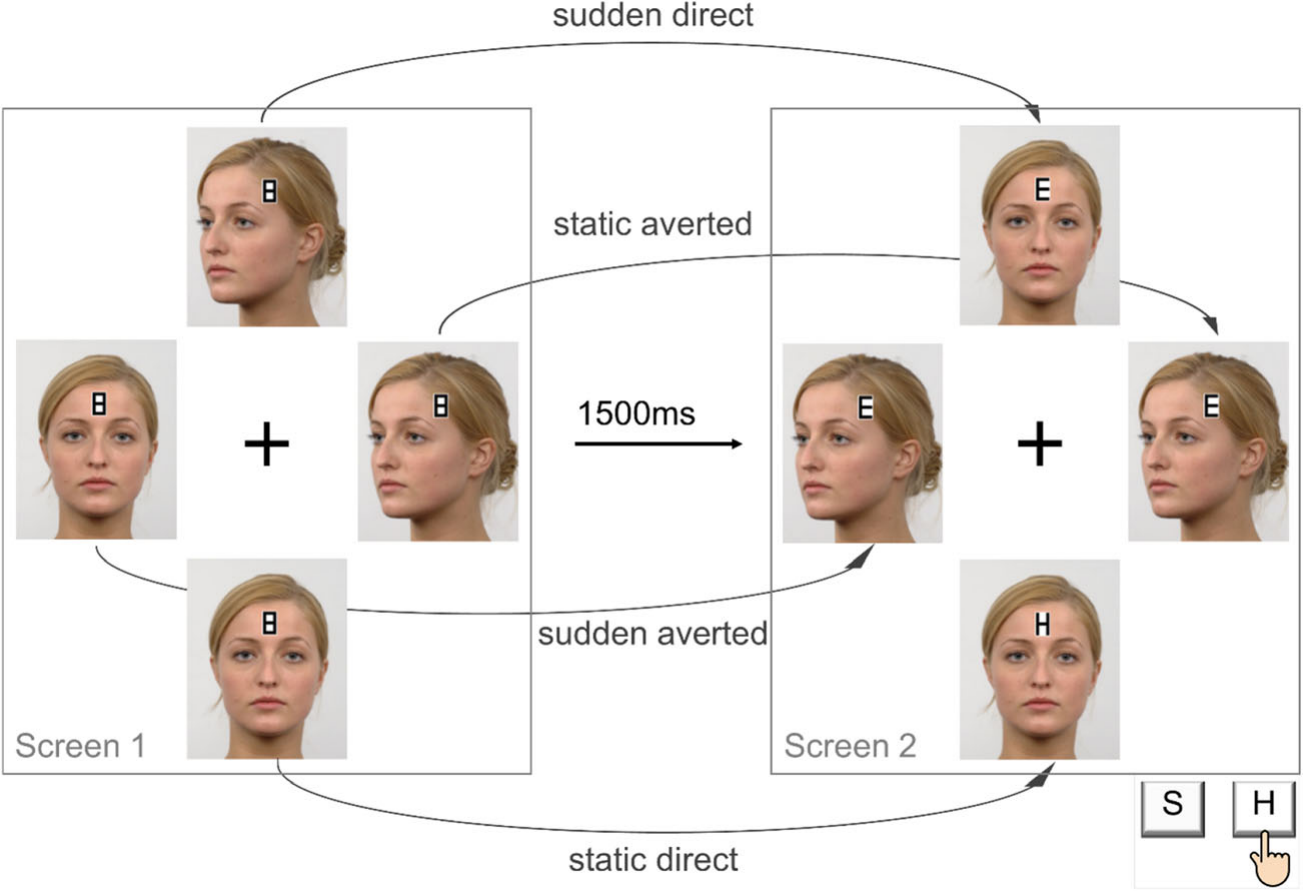
**S**ample trial sequence of Experiment 1. Note. Number 8 figures overlaid the four stimuli in screen 1 and were replaced by one target and three distraction letters after 1,500 ms. Simultaneously, one direct stimulus changed to averted and one averted stimulus changed to direct while the other two stimuli remained unchanged, resulting in four experimental conditions. Participants were required to react as fast as possible to the target letter by pressing the corresponding response key. This set-up was kept for Experiments 1–6, but stimuli varied (see Fig. 2)

We employed the paradigm of Böckler et al. (2014) with six different stimulus sets. In the original experiment, participants saw two displays, each consisting of four images of the same face positioned around a central fixation cross. Participants were repeatedly instructed to keep their eyes fixated on this cross throughout the experiment. In the first display, two of the faces depicted direct gaze while the other two faces looked to the side. Each of the four faces had the number “8” positioned on their forehead. After 1,500 ms, the number 8 figures were replaced by three distractor letters (“E”/”U”) and one target letter (“H”/“S”) to which the participants were required to respond by pressing “H” or “S” with the index fingers of both hands on a keyboard. Simultaneous to target presentation, two of the faces changed their orientation: one direct face suddenly changed to averted (*sudden-averted*) and one averted face suddenly looked straight ahead (*sudden-direct*). The other two faces remained static (*static-direct; static-averted*). Across 384 trials, identity and position of the target and distraction letters as well as locations of gaze and motion cues appeared equally often in all possible combinations. A sample trial sequence with photographic face stimuli from our Experiment 1 is displayed in Fig. 1.

### Participants

The number of participants for each experiment was determined using G*power3 (Faul et al., 2007), assuming 80% power and an α of .05 with a small effect size, resulting in 34 participants for each experiment. In sum, we tested 204 participants with normal or corrected-to-normal vision (Table 1). All participants gave informed consent and were compensated with 7€ or course credit. The present study complies with the ethical standards of the 1964 Declaration of Helsinki regarding the treatment of human participants.

**Table 1:** Data exclusions and gender, age, and handedness of participants in Experiments 1*–*6

Experiment	Total N I final sample	Excluded due to mean error rate +2SD	Females	Mean age, y (SD)	Right-handed
1	33	1	25	22.23 (±3.22)	25
2	32	2	24	23.91 (±3.60)	27
3	32	2	26	27.10 (±9.11)	31
4	33	1	22	22.45 (±2.61)	32
5	32	2	24	24.31 (±4.85)	30
6	32	2	26	23.91 (±4.01)	29

### Stimuli

**Fig. 2:**
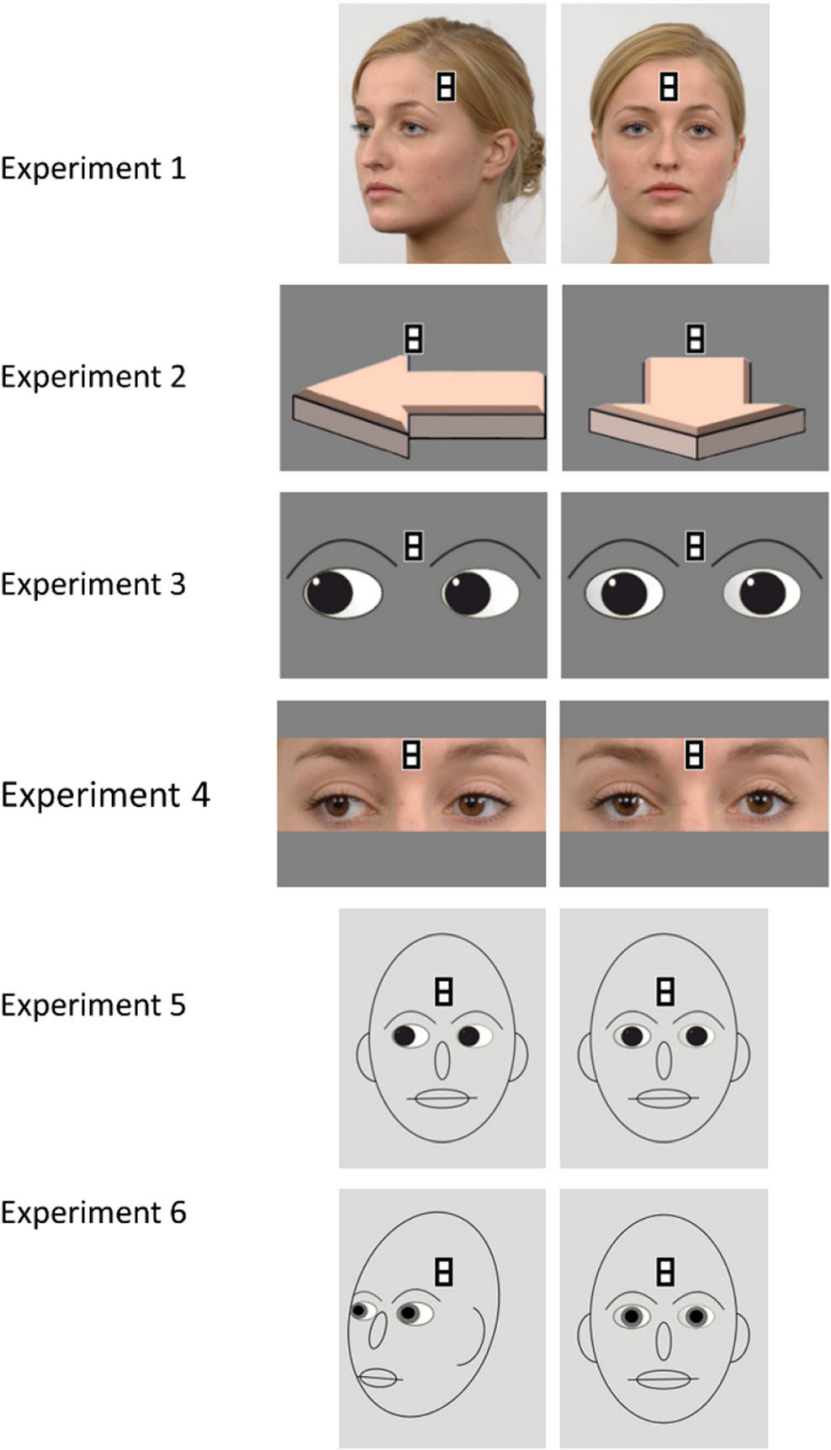
**S**timuli for Experiments 1*–*6. Note. Experiment 1: images of real faces (replication). Experiment 2: arrows (no social but directional information). Experiment 3: schematic eyes (no photographic and no holistic context). Experiment 4: images of real eyes (photographic social information; no holistic context). Experiment 5: schematic face without head turn (no photographic social information; holistic context). Experiment 6: schematic face with head turn (no photographic social information; holistic context)

Stimuli of all experiments are displayed in Fig. 2. We chose one female face from the Radboud Face Database (RaFD) (Langner et al., 2010) for Experiment 1 that we showed either in a direct or an averted position. The images were 200 × 250 pixels (1.21 × 1.52° of visual angle). To investigate whether directional, symbolic signals are sufficient for attention capture, we used arrows in Experiment 2. In Experiments 3 and 4, we employed isolated eyes to address the necessity of a holistic facial context. The eyes in Experiment 4 also stem from the RaFD. For Experiment 3, we designed schematic eyes on the basis of images from the RaFD.

The eye regions of the same images were used for Experiment 4. For both experiments, the images were 259 × 180 pixels (1.57 × 1.09° of visual angle). For Experiment 5, we inserted the comic-style eyes from Experiment 3 into a schematic facial configuration that was based on the images of the *direct* condition of experiment 1. However, analogous to Experiments 3–4, we kept frontal head orientation for averted stimuli, hence constricting the illusory motion to the area of the eyes. In Experiment 6, we took the comic-style faces one step closer to the photographic faces of Experiment 1 by rotating the averted-stimulus by 45° to create the impression of a head-turning movement and by adding pupils to the eyes. Across all experiments, we devoted special attention to keeping all relevant aspects of the stimuli as similar as possible.

### Analyses

Reaction time (RT) was defined as the time window from target onset until the first key press. In each experiment, participants with error rates +2SD above the global mean were removed. In the remaining data sets, RTs ±2SD of the participant’s mean in each condition and all RTs of trials that were associated with errors were excluded from further analysis. Table 2 provides an overview of error and exclusion rates as well as mean RTs for each condition of each experiment. RTs of all experiments are visualized in Fig. 2. All data were submitted to two 2 × 2 × 6 mixed-effects ANOVAs with the within-subject factors motion (static, sudden) and gaze/direction (direct, averted) and the between-subject factor experiment (1–6), entering mean RTs and error rates as dependent variables. Differences between experiments were further investigated with individual ANOVAs for each experiment. Finally, for each of Experiments 2–6, an ANOVA on RTs with the between-subjects factor *experiment* was conducted to compare it to Experiment 1. For better interpretation of null results, we drew on Bayesian statistics in addition to traditional null-hypothesis testing. In each case of a non-significant gaze/direction or motion effect, we performed Bayesian t-tests to calculate non-directional Bayes factors (BFs) with a prior distribution value of 1. Following Rouder et al. (2009), BFs were computed as *ƒ* (data | H_0_) /*ƒ* (data | H_1_) and interpreted as evidence for the null hypothesis when BF > 3 or as evidence for the alternative hypothesis when BF < 1/3.

**Table 2:** Reaction times (RTs), error rates, and exclusion rates across Experiments 1–6

Experiment	Condition										Exclusion rate
	Total		Sudden direct		Static direct		Sudden averted		Static averted		
	Mean RT (SD)	Mean error rate (SD)	Mean RT (SD)	Mean error rate (SD)	Mean RT (SD)	Mean error rate (SD)	Mean RT (SD)	Mean error rate (SD)	Mean RT (SD)	Mean error rate (SD)	
1 (photographic faces)	1002 (±123)	3.67 (±3.03)	934 (±104)	2.72 (±1.84)	1005 (±126)	3.73 (±3.50)	1009 (±109)	4.14 (±2.89)	1061 (±123)	4.1 (±3.49)	7.18
2 (arrows)	1006 (±115)	2.9 (±2.33)	982 (±112)	2.64 (±2.75)	1025 (±117)	2.83 (±2.17)	981 (±106)	2.67 (±2.23)	1037 (±120)	3.45 (±2.14)	6.25
3 (schematic eyes)	1003 (±139)	3.1 (±2.84)	983 (±139)	3.16 (±2.94)	989 (±136)	3.48 (±3.05)	989 (±136)	2.54 (±2.47)	1011 (±136)	3.22 (±2.90)	6.3
4 (photographic eyes)	950 (±151)	3.18 (±2.65)	943 (±151)	3.09 (±2.82)	960 (±147)	3.50 (±2.87)	932 (±146)	2.97 (±2.32)	965 (±163)	3.16 (±2.66)	7.44
5 (schematic faces without head turn)	952 (±130)	3.72 (±2.83)	937 (±114)	3.48 (±3.10)	962 (±136)	3.74 (±2.98)	941 (±137)	3.55 (±2.49)	969 (±136)	4.10 (±2.8)	8.14
6 (schematic faces with head turn)	987 (±215)	3.52 (±2.78)	986 (±230)	3.84 (±2.78)	998 (±207)	2.77 (±2.33)	975 (±218)	3.48 (±2.73)	990 (±213)	4.00 (±3.19)	8.24

*Note*. Mean RTs of correct responses in ms; error and exclusion rates in %

Considering the non-normal distribution of data, we took an additional, alternative approach to statistical analysis. First, participant- and trial-wise exclusions were based on predefined threshold values instead of on means and SDs. Hence, in each experiment, data sets of participants who performed below or at chance (error rate ≥ 50%) were excluded from the analysis. In the remaining data sets, trials with RTs below 150 ms or above 2,500 ms were removed. In a second step, RTs were log transformed and entered into a 2 × 2 repeated-measures ANOVA with the within-subject factors motion (static, sudden) and gaze/direction (direct, averted) for each experiment individually. In a similar way to our original analysis, Bonferroni-corrected *t-*tests were applied to resolve interaction effects and non-directional Bayes factors were calculated for non-significant main effects of gaze or motion. The results of this analysis, which revealed a highly similar pattern as the results described in the following section, can be found in Appendix A.

## Results and discussion

**Fig. 3:**
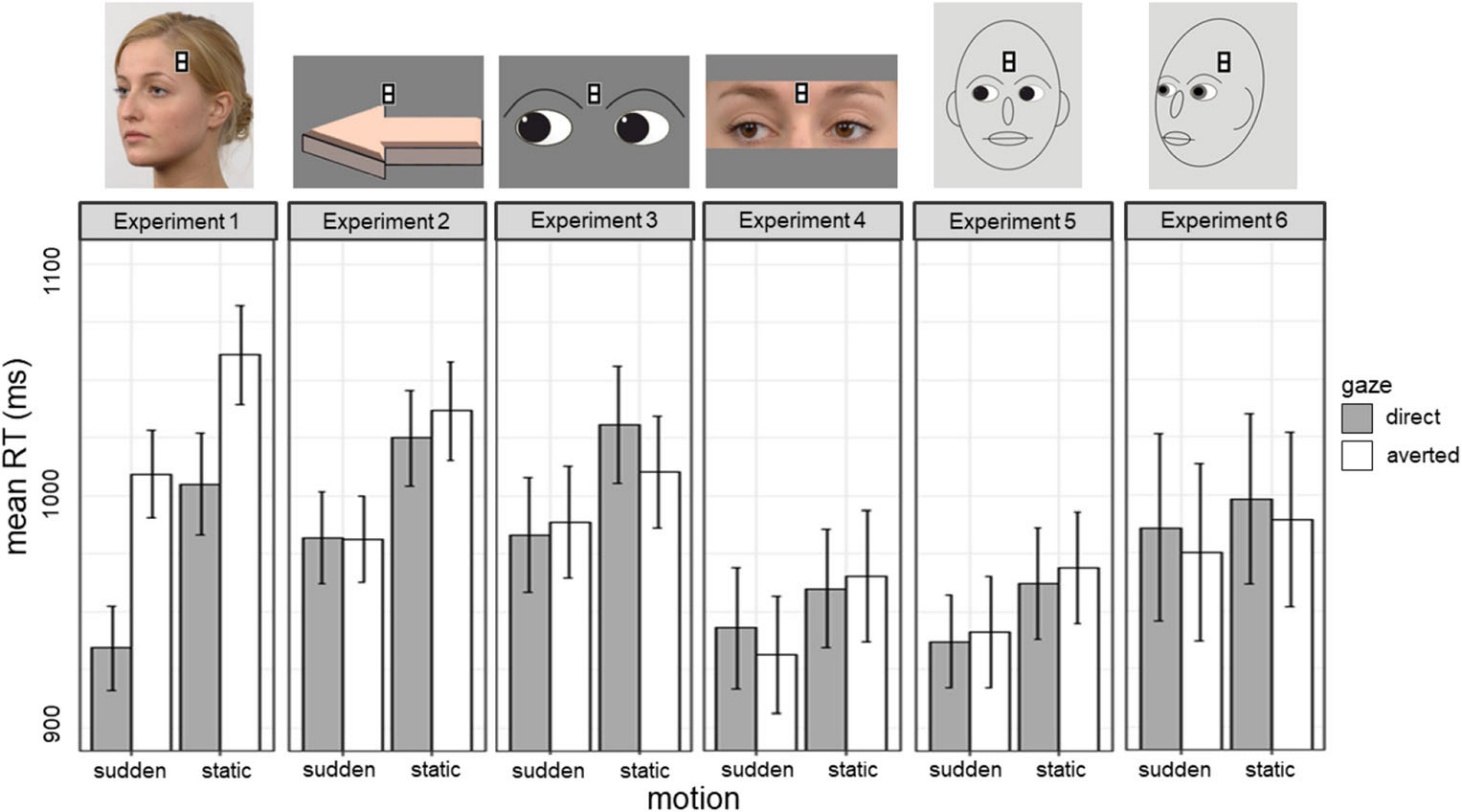
**M**ean reaction times (RTs) for all conditions for each of Experiments 1–6. Note**.***Mean RTs for targets appearing on stimuli directed towards participants are presented in grey; meanRTs for targets appearing on averted stimuli are depicted in white. Error bars represent standard errors*

**Fig. 4:**
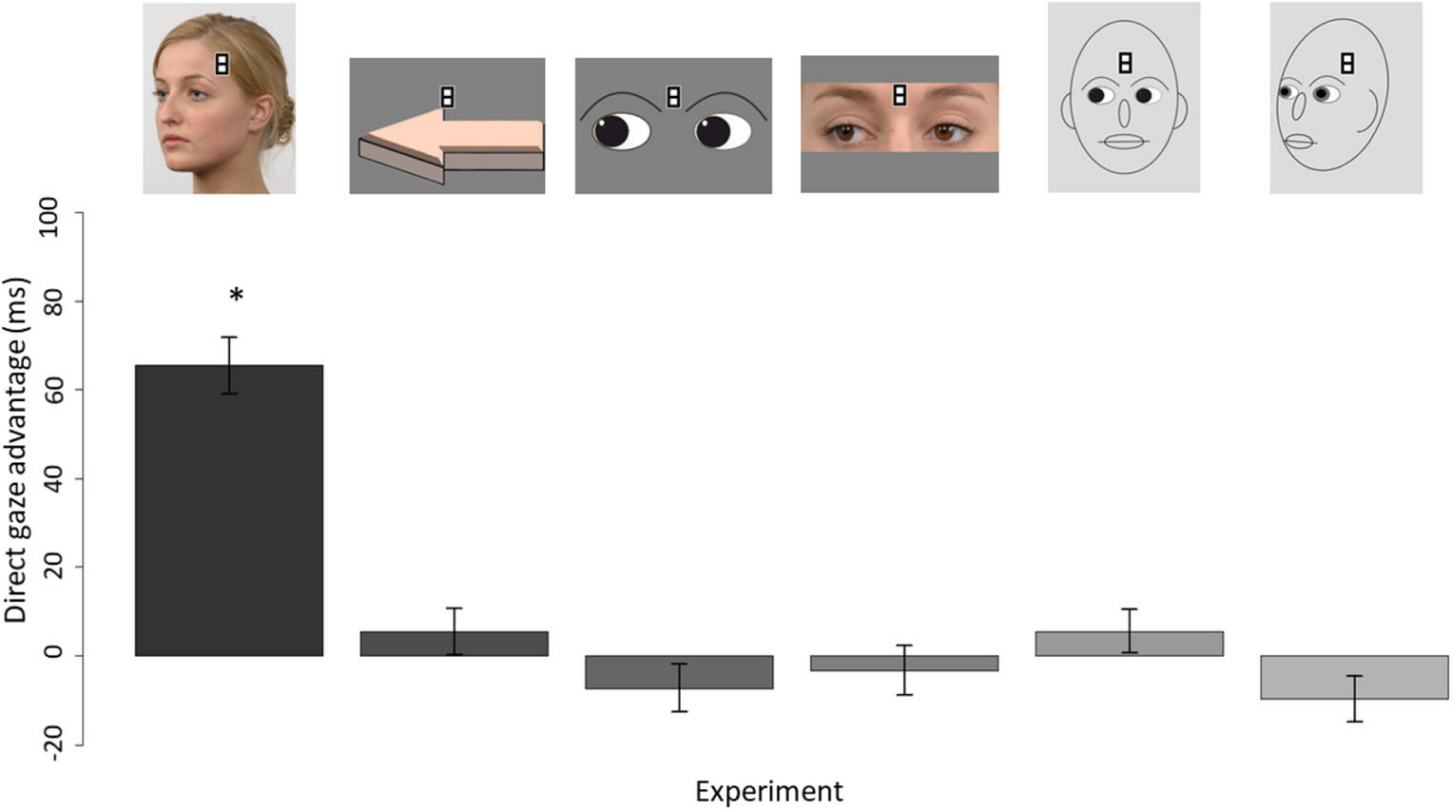
**D**irect gaze/direction advantage for each of Experiments 1–6. Note**.** Direct gaze/direction advantage calculated as mean reaction time (RT) of correct responses for targets appearing on averted stimuli – mean RT of correct responses for targets appearing on direct stimuli. Error bars represent standard errors

The mean RTs of each combination of gaze and motion for each of Experiments 1–6 are displayed in Fig. 3. The size of the direct-gaze effect for each experiment is visualized in Fig. 4. The data sets that the following analyses are based on are available in the Open Science Framework (DOI: 10.17605/OSF.IO/2JZGS). None of the experiments was preregistered.

### Omnibus analysis

To test for overall differences between experiments, we performed an omnibus analysis by entering the mean correct RTs of all six experiments with the between-subjects factor experiment (1, 2, 3, 4, 5, and 6) and the within-subject factors gaze/direction (direct, averted) and motion (static, sudden) to a mixed-effects ANOVA. We found a significant gaze/direction effect across all experiments (*F*(1, 188) = 15.87, *p* = .001, η^2^ = .001) as well as a motion effect (*F*(1, 188) = 82.96, *p* < .001, η^2^ = .014). Hence, overall, participants responded faster to targets appearing on stimuli that were directed towards them compared to away from them, and to targets appearing on stimuli that changed direction simultaneous to target presentation. Critically, the interaction effect of gaze/direction × experiment (*F*(5, 188) = 24.28, *p* < .001, η^2^ = .008) was also significant, indicating that the size of the gaze/direction effect differed substantially between the six experiments. We also found a small two-way interaction between motion × experiment (*F*(5, 188) = 3.45, *p* =.005, η^2^ = .003) and a small three-way interaction (*F*(5, 188) = 2.835, *p* =.017, η^2^ = .001), suggesting a modulation of the motion effect as well as a modulation of the interplay between gaze/direction and motion by experiment. Performing the same analysis with error rates as a dependent variable revealed a significant main effect of motion (*F*(1, 188) = 5.41, *p* = .021, η^2^ = .003), indicating more errors for static stimuli, in line with RT results. No other effects were significant (all *p*s > .05). Exact *p*-values and effect sizes for all non-significant effects are reported in Table 3 in Appendix B.

To disentangle interaction effects of this initial omnibus analysis, individual analyses on RTs were performed for each experiment and results are reported in the following.

### Experiment 1: Photographs of real faces

As in the original study (Böckler et al., 2014), RTs were shorter when targets were presented on a face with direct gaze compared to averted gaze (*F*(1, 32) = 84.11, *p* < .001, η^2^ = .076). We also found a significant motion effect with shorter RTs in the *sudden* condition (*F*(1, 32) = 17.80, *p* < .001, η^2^ = .067). In line with RT results, participants produced more errors in response to averted compared to direct faces, as evident in a significant main effect of gaze on error rates (*F*(1, 32) = 6.87, *p* = .013, η^2^ = .023). No other effects were significant (all *p*s > .05).

This pattern of results replicates earlier findings of attention capture by direct gaze.

### Experiment 2: Arrows

In contrast to Experiment 1, the main effect of gaze/direction was not significant (F(1,31) = 0.902, *p* = .350; BF_01_ = 3.5). The main effect of motion was significant, with shorter RTs for moving stimuli (*F*(1, 31) = 32.10, *p* < .001, η^2^ = .046). None of the other effects were significant for RTs and error rates (all *p*s > .05).

We conducted an ANOVA on RTs with the additional between-subjects factor experiment (1, 2) to systematically test for differences between the gaze/direction effects for pictures of faces and arrows. Besides significant overall effects for gaze/direction (*F*(1, 61) = 60.31, *p* < .001, η^2^ = .022) and motion (*F*(1, 61) = 52.71, *p* < .001, η^2^ = .016), we found a significant gaze/direction × experiment interaction (*F*(1, 61) = 42.52, *p* = .001, η^2^ = .016), emphasizing the difference between magnitudes of the gaze/direction effect between naturalistic face stimuli and arrows. The three-way interaction effect of gaze/direction × motion × experiment was also significant (*F*(1, 61) = 6.75, *p* .012, η^2^ = .002). Because the two-way interaction of gaze/direction and motion was not even close to significance in either of the individual experiments, we refrained from further analyzing this interaction. No other effects were significant (all *p*s > .05).

These results indicate that being pointed at by a directional symbolic stimulus such as an arrow does not capture attention.

### Experiment 3: Schematic eyes

The main effect of gaze/direction was not significant (F (1,31) = 1.832, *p* = .186; BF_01_ = 2.36). We found a significant main effect of motion (*F*(1, 31) = 24.79, *p* < .001, η^2^ = .016), with faster RTs to targets appearing on moving stimuli. The gaze × motion interaction effect was significant (*F*(1, 31) = 6.49, *p* = .016, η^2^ = .002), with faster reactions to targets appearing on averted stimuli compared to direct stimuli in the static condition, but not in the sudden condition (static: *t*(31) = 2.504, *p* = .035; sudden: *p* = .802). No other effects were significant in RTs or error rates (all *p*s > .05).

The ANOVA with the additional between-subjects factor experiment (1, 3) revealed significant main effects of gaze (*F*(1, 63) = 42.27, *p* < .001, η^2^ = .013) and motion (*F*(1, 61) = 34.81, *p* < .001, η^2^ = .035) as well as a significant gaze × experiment interaction (*F*(1, 61) = 65.87, *p* = .001, η^2^ = .020), emphasizing the difference in the direct-gaze effect between real faces and schematic eyes. The three-way interaction of gaze × motion × experiment also reached significance (*F*(1, 61) = 9.44, *p* = .003, η^2^ = .002), reflecting the presence of a gaze × motion interaction for schematic eyes, which was absent for faces. No other effects were significant (all *p*s > .05).

These results suggest that eye gaze without photographic facial context information is insufficient to trigger the direct gaze advantage.

### Experiment 4: Photographs of real eyes

We found the typical main effect of motion on RTs (*F*(1, 32) = 12.96, *p* = .001, η^2^ = .007), but no main effect of gaze (*F* (1,32) = 0.302, *p* = .586; BF_01_ = 4.73)*.* No other effects were significant (all *p*s > .05).

Adding the between-subjects factor experiment (1, 4), we found significant main effects of gaze (*F*(1, 64) = 45.03, *p* < .001, η^2^ = .014) and motion (*F*(1, 64) = 28.81, *p* < .001, η^2^ = .026), as well as a significant interaction effect of gaze × experiment (*F*(1, 64) = 54.93, *p* < .001, η^2^ = .016), supporting the difference in gaze effects between photographs of faces and eyes. In addition, there was a motion × experiment interaction (*F*(1, 64) = 4.94, *p* = .030, η^2^ = .016) due to a smaller motion effect for photographs of eyes compared to faces, and a gaze × motion × experiment interaction (*F*(1, 61) = 6.06, *p* = .017, η^2^ = .001). No other effects were significant (all *p*s > .05).

These findings further emphasize that the presentation of a holistic face is vital for the direct-gaze effect. We found no statistically significant gaze effect for photographs of real eyes and the BF provided substantial evidence for the null hypothesis. This conclusion is in line with Böckler et al. (2015) reporting a collapse of the (sudden) direct-gaze effect when the integration of eye and face was disrupted by presenting faces upside down.

### Experiment 5: Schematic faces without head-turn

Again, we found a significant main effect of motion on RTs (*F*(1, 31) = 19.18, *p* < .001, η^2^ = .010) and no attention capture effect for direct gaze (F (1,31) = 1.22, *p* = .278; BF_01_ = 3.42). No other effects were significant (all *p*s > .05).

Adding the between-subjects factor experiment (1, 5), we found significant main effects of gaze (*F*(1, 63) = 65.55, *p* < .001, η^2^ = .021) and motion (*F*(1, 63) = 30.45, *p* < .001, η^2^ = .031). Again, we found a significant interaction of gaze × experiment (*F*(1, 63) = 46.70, *p* < .001, η^2^ = .015), indicating that the gaze effect was substantially larger for photographic face stimuli. There was a small interaction of gaze × motion in this ANOVA (*F*(1, 63) = 4.77, *p* = .033, η^2^ = .005), suggesting that the motion effect was also larger for photographs of real faces than for schematic faces. No other effects were significant (all *p*s > .05).

Revealing no direct-gaze effect in schematic faces, these findings further emphasize the relevance of naturalistic social cues for the direct gaze advantage.

### Experiment 6: Schematic faces with head-turn

No significant effects were found for Experiment 6 (all *p*s > .05; gaze: *F*(1,31) = 3.623; *p* = .066; BF_01_ = 1.09; motion: *F*(1,31) = 1.667; *p* = .206; BF_01_ = 2.54).

When adding the between-subjects factor experiment (1, 6), we found significant main effects of gaze (*F*(1, 63) = 40.06, *p* < .001, η^2^ = .007) and motion (*F*(1, 63) = 17.33, *p* = .003, η^2^ = .012) as well as a significant gaze × experiment interaction effect (*F*(1, 63) = 72.712, *p* < .001, η^2^ = .01), suggesting a gaze effect for photographic face stimuli only. The experiment × motion interaction was also significant (*F*(1, 63) = 7.13, *p* = .01, η^2^ = .005) because the motion effect was absent for schematic and turning faces. No other effects were significant (all *p*s > .05).

The absence of a direct gaze advantage in schematic faces with head-turns further supports the necessity of naturalistic social stimuli for the direct-gaze effect. In addition, we found an absence of a motion effect for the schematic faces with head-turn. Given that the head-turn orientation in schematic faces was kept identical to that in the original photographic faces (Experiment 1), the absence of a motion effect is not due to a mere reduction of the extent of motion per se. Nonetheless, our constructed schematic faces did not elicit apparent motion effects, even though the same faces without head-turn did (Experiment 5).

## General discussion

The present study investigated the aptitude of various ostensive stimuli to capture attention. Six experiments systematically manipulated the degree of photographic and holistic social context information and assessed the (sudden) direct-gaze effect. Specifically, we compared (1) photographic human faces, (2) arrows, (3) schematic, and (4) photographic isolated eye stimuli, and schematic face stimuli (5) without or (6) with head turn.

Firstly, results of Experiment 1 revealed a reliable direct-gaze effect for human faces, replicating prior studies (Böckler et al., 2014; Boyer & Wang, 2018) and substantiating the notion of an exceptional processing of direct gaze cues (Senju & Johnson, 2009). In contrast, no attention capture was found for symbolic self-directed cues (arrows, Experiment 2). This finding is somewhat surprising given that spatial cueing is reliably observed for arrows (Daum & Gredebäck, 2011), but fits the notion that reflexive attention to arrows and to biologically relevant gaze cues are based on distinct neural systems (Ristic et al., 2002).

Interestingly, we found no direct-gaze effect for schematic or photographic eyes outside of a facial context. These results seem at odds with some previous findings of processing advantages for direct over averted gaze with eyes-only stimuli (Chen & Yeh, 2012; Conty et al., 2006; Greene et al., 2011; Hayward & Ristic, 2015; Senju et al., 2005). However, this discrepancy can be accounted for when taking a closer look at the tasks. In two of the abovementioned studies (Conty et al., 2006; Senju et al., 2005), participants were asked to detect direct gaze stimuli among averted gaze distractors as quickly as possible. Hence, while gaze was a distractor in our task, it was the target in these experiments. Without task-driven requirements to process gaze characteristics, isolated eye stimuli in our experiment may not have been encoded sufficiently to capture attention. Furthermore, results of Conty et al. (2006) indicate another critical mediator of gaze processing: Even for eyes-only stimuli, direct gaze was more salient than averted gaze when the visible part of the face (the region around the eyes) was oriented in the same direction as the pupils. This aspect was lacking in our averted eyes-only stimuli (see Fig. 1). Note, however, that congruency between head and eye orientation was not sufficient to elicit a direct-gaze effect with schematic face stimuli in our study (Experiment 6), indicating that naturalness of the stimuli is a further prerequisite for the direct-gaze effect as investigated with our paradigm. Finally, a critical difference between our paradigm and spatial cueing paradigms, such as in Hayward and Ristic (2015), is the location of cue presentation within the visual field: While cues are usually presented centrally and hence overtly fixated in spatial cueing, participants in our experiments were explicitly instructed to fixate on a centrally presented cross throughout the task so that cues would appear in the periphery and be covertly attended. This difference can have a substantial impact on (social) attention (Boyer & Wang, 2018; Riechelmann et al., 2020). Perception is most accurate and contrast-sensitive in the foveal region and decreases from the central-to-peripheral gradient of the visual field (Burnat, 2015; Kitterle, 1986). While face processing is particularly impressive even at high eccentricities (Hershler et al., 2010), it remains unclear whether this processing advantage extends onto isolated eyes and, hence, specific investigations of related effects are necessary.

Remarkably, the gaze-cueing paradigm robustly produces orientation effects with numerous stimulus types, including photographic and schematic face stimuli, both upright and inverted (Tipples, 2005), faces with strabismus (Hietanen & Yrttimaa, 2005) and even arrows (Ristic et al., 2002). Within this paradigm, systematic investigation of facial feature information indicated that local processing of the eyes has a major impact on reflexive orienting (Frischen et al., 2007; Tipples, 2005). It might be worthwhile to conduct similar investigations with the attention capture paradigm to assess systematically the impact of local feature information on the sudden direct-gaze effect. From what we know, it appears that the attention capture paradigm, in contrast to that of spatial cueing, requires a holistic facial context that allows for a meaningful interpretation of the embedded eyes. In previous experiments, images of realistic faces elicited a direct-gaze effect even when only the eyes were moving (van der Wel et al., 2018), and that this effect collapsed when the eyes were closed or when the integration of eyes and face was disrupted by inverting face stimuli (Böckler et al., 2015). However, visibility of the eyes was not strictly necessary: The effect still occurred when eyes were covered with opaque sunglasses. Taken together, these findings indicate that, instead of being drawn by direct gaze per se, our preferential attention to the ostensive signal of “being addressed by someone” has an impact on attention capture (Csibra & Gergely, 2009).

In contrast to the pronounced direct-gaze effect for the more realistic photographic face stimuli, there was no indication of such an effect for schematic facial configurations, irrespective of head orientation. One explanation is that the naturalness of the stimuli is a further crucial factor for direct gaze advantages (Hamilton, 2016). Naturalistic stimuli convey a larger potential to interact socially, and the mere opportunity to do so can alter social attention (Laidlaw et al., 2011; but see also Riechelmann et al., 2019). A promising line of future research may be to gradually and independently manipulate the degrees of naturalness of stimuli (e.g., by employing avatars) and of the interaction opportunity (e.g., by employing real or virtual reality setups (see Rubo et al., 2020)), in order to tackle the minimum requirements and relative contribution of realistic face information and interaction opportunity for attention capture by direct gaze.

Taken together, we replicated the direct-gaze effect for photographic faces, but did not find an effect of gaze/arrow direction in any other stimulus configuration. While we are aware that the interpretation of null results is tricky, further results support our conclusions. First, between-experiment ANOVAs revealed significant differences between direct-gaze effects of Experiment 1 and all other experiments. Second, numerical differences between averted minus direct stimuli in Experiments 2–6 are also negligible (maximum 3 ms), which suggests that the absence of significant effects is not due to mere power issues. Finally, frequentist inferences of non-significant gaze effects were supported by Bayesian results with none of the BFs in Experiments 2–6 providing evidence for the alternative hypothesis (no BF < 1/3). For three experiments, namely Experiments 2, 4, and 5, the BFs provided clear evidence for the null hypothesis (BF > 3), while for experiments with ambiguous evidence (Experiments 3 and 6), RTs were slightly faster for *averted* stimuli rather than for direct stimuli. Of course, further research is necessary to strengthen the absence of direct-gaze effects in reduced social stimuli and to further explore the boundary conditions and underlying factors of this absence.

Although we varied our stimulus set along several dimensions, performance for targets on moving stimuli was generally better than on stimuli that remained static. In other words, except for Experiment 6, the motion effect remained present across the degree of social information, the degree to which this information was naturalistic, and the degree to which isolated features versus holistic faces were presented. This pattern confirms the finding that sudden-onset motion captures attention (Abrams & Christ, 2005) and demonstrates generalizability to a variety of stimuli. In this light, the finding that the motion effect in schematic faces (Experiment 5) vanishes when a head-turn movement is introduced (Experiment 6) is particularly surprising. One possibility is that motion effects are not as stable as generally assumed. However, considering findings from Experiments 1–5 and the vast body of literature on motion effects, including previous studies using the same paradigm (Böckler et al., 2015; Byer et al., 2018), this seems rather unlikely. An alternative explanation is that other attentional or volitional processes cancelled out motion effects in Experiment 6. That is, basic or configural aspects of the head-turn stimuli might have prevented them from inducing an apparent motion effect. Critically, findings from Bayes analyses on motion effects in Experiment 6 were ambiguous, indicating that further research is necessary to replicate and potentially clarify these (null-) effects.

## Conclusions

To conclude, the present results indicate that even though attention capture by direct gaze is triggered by small stimulus facets such as the eyes, it critically depends on facial context information. Direction information that was conveyed by symbolic stimuli, by isolated eyes, or even by schematic faces with head-turns identical to those in photographs did not catch attention in a similar manner. This pattern speaks to the idea that social context information has an early effect on attention capture and can modulate our subsequent perception, cognition and interaction (Laidlaw et al., 2011). Hence, instead of blindly being drawn by gaze wherever we spot it, we may only catch the eye of someone we can potentially interact with.

## Appendix A

### Alternative analyses

The following alternative analyses were based on using different data-trimming decisions and log-transformed RT data (see Analyses section in the article for details).

Results of Experiment 1: Photographs of real faces

Trial-wise exclusion rate: 4.31%

ANOVA

**Table:**

Effect	df	*F*	*p*	<.05	η^2^
Motion	1,33	22.758	0.000	*	0.067
Gaze	1,33	91.573	0.000	*	0.077
Motion:gaze	1,33	3.069	0.089		0.002

**Results of Experiment 2: Arrows**

Trial-wise exclusion rate: 3.36%

ANOVA

**Table:**

Effect	df	*F*	*p*	<.05	η^2^
Motion	1,31	32.541	0.000	*	0.049
Direction	1,31	0.034	0.854		0.000
Motion:direction	1,31	0.964	0.343		0.000

Direction: BF_01_ = 5.211

**Results of Experiment 3: Schematic eyes**

Trial-wise exclusion rate: 4.74%

ANOVA

**Table:**

Effect	df	*F*	*p*	<.05	η^2^
Motion	1,33	21.481	0.000	*	0.013
Gaze	1,33	0.949	0.337		0.000
Motion:gaze	1,33	4.625	0.039	*	0.002

Gaze: BF_01_ = 3.514

Paired t-test (Bonferroni-corrected)

**Table:**

Level	df	*t*	*p*	<.05	method
Sudden	33	-0.810	.848		paired
Static	33	2.284	.058		paired

**Results of Experiment 4: Photographs of eyes**

Trial-wise exclusion rate: 4.35%

ANOVA

**Table:**

Effect	df	*F*	*p*	<.05	η^2^
Motion	1,33	22.547	< .001	*	0.011
Gaze	1,33	0.000	0.995		0.000
Motion:gaze	1,33	0.460	0.502		0.000

Gaze: BF_01_ = 5.442

**Results of Experiment 5: Schematic faces without head-turn**

Data exclusion rate: 5.09%

ANOVA

**Table:**

Effect	df	*F*	*p*	<.05	η^2^
Motion	1,33	13.122	0.001	*	0.006
Gaze	1,33	1.759	0.194		0.001
Motion:gaze	1,33	0.058	0.812		0.000

Gaze: BF_01_ = 2.442

**Results of Experiment 6: Schematic faces with head-turn**

Trial-wise exclusion rate: 5.33%

ANOVA

**Table:**

Effect	df	*F*	*p*	<.05	η^2^
Motion	1,33	1.662	0.206		0.001
Gaze	1,33	2.046	0.162		0.000
Motion:gaze	1,33	0.001	0.982		0.000

Motion: BF_01_ = 2.550

Gaze: BF_01_ = 2.151

### Appendix B

**Table 3:** P-values and effect sizes of non-significant effects for Experiments 1–6

Experiment	Dependent variable	Independent variables	Effect	*p-*value	η^2^
1–6	RT	Experiment, gaze, motion	Experiment	.392	.026
1–6	RT	Experiment, gaze, motion	Gaze × motion	.651	.000
1–6	Error rate	Experiment, gaze, motion	Experiment	.567	.013
1–6	Error rate	Experiment, gaze, motion	Gaze	.137	.001
1–6	Error rate	Experiment, gaze, motion	Gaze × motion	.397	.001
1–6	Error rate	Experiment, gaze, motion	Experiment × motion	.550	.003
1–6	Error rate	Experiment, gaze, motion	Experiment × gaze	.058	.007
1–6	Error rate	Experiment, gaze, motion	Experiment × motion × gaze	.246	.005
1	RT	Gaze, motion	Gaze × motion	.077	.002
2	RT	Gaze, motion	Gaze	.350	.001
2	RT	Gaze, motion	Gaze × motion	.203	.001
1,2	RT	Experiment, gaze, motion	Experiment	.821	.001
1,2	RT	Experiment, gaze, motion	Experiment × motion	.255	.002
1,2	RT	Experiment, gaze, motion	Gaze × motion	.408	.000
3	RT	Gaze, motion	Gaze	.186	.001
1,3	RT	Experiment, gaze, motion	Experiment	.973	.000
1,3	RT	Experiment, gaze, motion	Experiment × motion	.108	.003
1,3	RT	Experiment, gaze, motion	Experiment × motion × gaze	.671	.000
4	RT	Gaze, motion	Gaze	.586	.001
4	RT	Gaze, motion	Gaze × motion	.108	.001
1,4	RT	Experiment, gaze, motion	Experiment	.103	.037
1,4	RT	Experiment, gaze, motion	Gaze × motion	.846	.000
5	RT	Gaze, motion	Gaze	.278	.000
5	RT	Gaze, motion	Gaze × motion	.788	.000
1,5	RT	Experiment, gaze, motion	Experiment	.091	.040
1,5	RT	Experiment, gaze, motion	Gaze × motion	.258	.000
1,5	RT	Experiment, gaze, motion	Experiment × motion × gaze	.135	.001
6	RT	Gaze, motion	Motion	.206	.001
6	RT	Gaze, motion	Gaze	.066	.001
6	RT	Gaze, motion	Gaze × motion	.840	.000
1,6	RT	Experiment, gaze, motion	Experiment	.717	.002
1,6	RT	Experiment, gaze, motion	Gaze × motion	.253	.000
1,6	RT	Experiment, gaze, motion	Experiment × motion × gaze	.155	.000

## Declarations

### Availability of data and materials

The data sets analyzed during this study are available in the Open Science Framework (DOI: 10.17605/OSF.IO/2JZGS). None of the experiments was preregistered.

### Code availability

The codes for stimulus presentation and data analysis are available from the corresponding author upon request.
